# The antiproliferative effect of C_2_-ceramide on lung cancer cells through apoptosis by inhibiting Akt and NFκB

**DOI:** 10.1186/1475-2867-14-1

**Published:** 2014-01-06

**Authors:** I-Ling Lin, Han-Lin Chou, Jin-Ching Lee, Feng-Wei Chen, Yao Fong, Wei-Chiao Chang, Hurng Wern Huang, Chang-Yi Wu, Wen-Tsan Chang, Hui-Min David Wang, Chien-Chih Chiu

**Affiliations:** 1 Department of Medical Laboratory Science and Biotechnology, Kaohsiung Medical University, Kaohsiung 807, Taiwan; 2 Department of Biotechnology, Kaohsiung Medical University, Kaohsiung 807, Taiwan; 3 Chest Surgery, Chi-Mei Foundation Medical Center, Yung Kang City, Tainan, 901, Taiwan; 4 Department of Clinical Pharmacy; Master Program for Clinical Pharmacogenomics and Pharmacoproteomics, School of Pharmacy, Taipei Medical University, Taipei, Taiwan; 5 Institute of Biomedical Sciences, National Sun Yat-Sen University, Kaohsiung 804, Taiwan; 6 Department of Biological Sciences, National Sun Yat-Sen University, 70 Lien Hai Road, Kaohsiung 804, Taiwan; 7 Division of Hepatobiliary and Pancreatic Surgery, Department of Surgery, Kaohsiung Medical University, Kaohsiung, Taiwan; 8 Department of Fragrance and Cosmetic Science; Graduate Institute of Natural Products, Kaohsiung Medical University, Kaohsiung 807, Taiwan

**Keywords:** Lung cancer, Apoptosis, NSCLC, Ceramides, p-Akt, p-NFκB, survivin, cyclin A2

## Abstract

The anticancer effects of ceramide have been reported in many types of cancers but less in lung cancer. In this study, we used C_2_-ceramide to further investigate its possible anticancer effects and mechanisms on non-small cell lung cancer (NSCLC) H1299 cells. The result of cell proliferation in terms of trypan blue assay showed high dose of C_2_-ceramide inhibited cell survival after 24 h treatment. The flow cytometry-based assays indicated the effect of apoptosis, chromatin condensation, and G_1_ arrest in terms of Annexin V/propidium iodide (PI), DAPI, and PI stainings, respectively. Moreover, the decreased protein level of p-Akt, p-NFκB, survivin and cyclin A2 were detected by Western blot assay. Taken together, these results indicated the antiproliferative effect of C_2_-ceramide is majorly responsible for cell apoptosis in lung cancer H1299 cells.

## Introduction

Approximately 80% of lung cancer belongs to non-small-cell lung cancer (NSCLC) and the other is SCLC histologically [1]. Although smoking is one of the main risk factors for lung cancer [2], about 10% patients are non-smokers [3]. NSCLC is generally detected and diagnosed at late stage and its prognosis is poor [4]. Therefore, the anticancer drugs for NSCLC treatment remain a challenge.

Akt, a serine/threonine kinase, regulates cell growth, cell cycle progression, survival and anti-apoptosis. Dysregulation of Akt was reported to be observed in various cancers including breast cancer [5] and lung cancer cells [6]. Furthermore, the constitutive activation of Akt has been shown to cause chemoresistant of cancer cells. Similarly, NFκB, an inflammatory-associated transcription factor, is also found to be constitutively activated or aberrantly expressed in lung cancer [7,8]. Therefore, targeting of Akt and NFκB signaling seems to be a promising strategy for lung cancer therapy [9-12].

Ceramides are important components composed of lipid molecules and can form into sphingolipids when added functional groups on their hydroxyl group such as phosphate choline or monosaccharide. When cells are triggered by certain stimuli, an enzyme called sphingomyelinase [13] would hydrolyze sphingolipids and cause the release of ceramides into cytoplasm, which can undergo many biological processes, such as differentiation, proliferation, growth arrest and apoptosis [14]. Ceramide was reported to act as an important mediator in apoptosis pathways. Exogenous ceramides have been demonstrated to induce anti-proliferation and apoptosis in many cancers [15]. Furthermore, accumulating evidence showed that ceramide inhibits proliferation of cancer cells via inhibiting Akt and NFκB signal pathways [16,17].

The ceramide-mediated anticancer effects have been reported in many types of cancers such as pancreatic [18], breast [19], gastric [20], hematologic [21] cancer. However, the final outcome of ceramide treatment may depend on the context of cell types. Previous studies showed that knockout of *NFκB p65* sensitizes embryonic fibroblasts toward C_2_-ceramide induced cell death [22]. On the contrary, the above study also found that C_2_-ceramide induces cell death and activation of NFκB in lung cancer H1299 cells [22]. The effects of C_2_-ceramide on apoptosis of H1299 cells were investigated previously [22,23]. In the current study, we examined the growth inhibitory property to NSCLC H1299 cells by C_2_-ceramide as well as its possible apoptosis mechanism, especially inhibiting Akt and NFκB pathways.

## Materials and methods

### Cell cultures

The H1299 lung cancer cells were maintained in DMEM medium (Invitrogen, Carlsbad, CA USA) containing 10% fetal bovine serum (FBS), 100 U/ml penicillin, 100 μg/ml streptomycin, 0.03% glutamine and 1 mM sodium pyruvate [24] and kept at 37°C in a humidified atmosphere with 5% CO_2_.

### Cell survival assay

Cell survival was determined by the trypan blue dye exclusion assay as previously described [25,26]. In brief, Cells were seeded at a density of 1 × 10^5^ cells per well. After 24 h of incubation, the cells were treated with C_2_-ceramide (#A7191, Sigma) at concentrations of 0, 10, 20, and 50 μM for 24 h, then 0.2% trypan blue were added to wells. Finally, the viable cells we are calculated by the Countess® Automated Cell Counter (Invitrogen, Diego, CA, USA). The assay was triplicated and the IC_50_ was calculated by the slope and intercept accordingly to two concentrations of C_2_-ceramide between the half-maximal proliferative inhibition.

### Apoptosis assay

Apoptosis was detected by annexin/PI staining (Pharmingen, San Diego, CA, USA) as previously described [27]. Briefly, cells were treated with C_2_-ceramide at concentrations of 0, 10, 20, and 50 μM for 24 h. After collection, cells were treated with 10 μg/ml of annexin V-fluorescein isothiocyanate and 5 μg/ml of PI for analysis with a FACSCalibur™ flow cytometer (Becton-Dickinson).

### Chromatin condensation assay

5 × 10^5^ H1299 cells were seeded onto a 6-well plate. After 24 h, cells were treated with indicated concentrations (0 to 50 μM) of ceramide for 24 h. After wards, cells were stained with 5 μg/ml of DAPI for 3 mins at 37°C. The level of chromatin condensation was determined by a flow cytometry (Becton-Dickinson). At least 10,000 stained cells were counted and calculated as percentage of chromatin condensation compared to those of the control cells.

### Cell cycle distribution

Propidium iodide (PI, Sigma, St. Louis, MO, USA) staining for DNA content measurement was performed as described previously [28]. Briefly, cells were treated with 0, 10, 20, and 50 μM of C_2_-ceramide for 24 h. After collection, cells were washed twice with PBS before 70% ethanol fixation. After centrifugation, the cells were incubated with 10 μg/ml PI and 10 μg/ml RNase A in PBS for 15 min at room temperature in the dark. Cell cycle analyses were performed using a FACSCalibur flowcytometer (Becton-Dickinson, Mansfield, MA, USA).

### Western blotting

Western blot assay was performed as described previously [27]. Briefly, cells were collected for lysate preparation. After centrifugation, and protein concentrations of lysates were determined. Protein lysates for 40 μg were loaded and electrophoresed by 10% SDS-polyacrylamide gel (PAGE) and then transferred to membrane. The membranes were blocked with 5% non-fat milk. Subsequently, it was reacted with primary antibodies against t-Akt (#1081), p-NFκB (Ser536, #2220) and Bax (#1063, Epitomics, CA, USA); t-NFκB (sc-8008), β-catenin (sc-7963) and p-Akt (Ser473, sc-7985, Santa Cruz Biotech, Santa Cruz, CA, USA); Cyclin A2 (GeneTex Co., Cat No. GTX103042); survivin (AnaSpec, San Jose, CA, USA) and β-actin (#sc-8432, Santa Cruz Biotech), and their corresponding secondary antibodies. The ECL™ (Amersham Piscataway, NJ, USA) chemiluminescence detection kit was applied.

### Statistical analysis

All data are presented as mean ± S.D. Comparison between experimental groups and vehicle controls was assessed by one-way ANOVA test.

## Results

### Anti-proliferative effects of C_2_-Ceramide-Treated H1299 lung cancer cells

**Figure 1:**
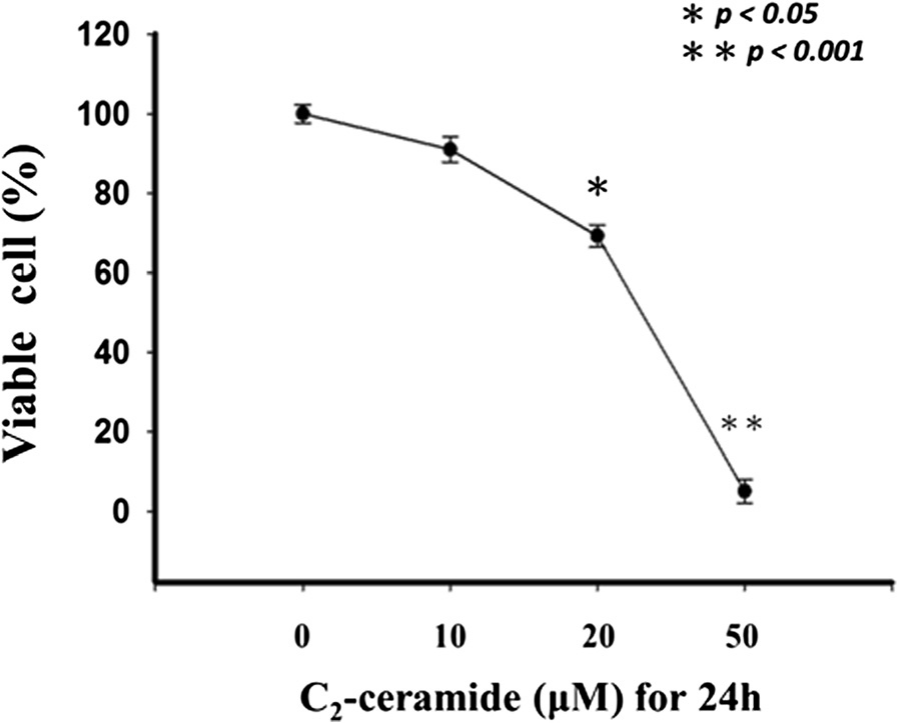
**Effect of C**_**2**_**-Ceramide on proliferation of H1299 cells.** The cell proliferation assay showed the inhibition of cell growth at high dose of treatment for 24 h.

In the trypan blue assay (Figure 1), the proliferation rates at various concentrations of C_2_-ceramide (0, 10, 20, and 50 μM) after 24 h were 100.0 ± 2.3, 90.1 ± 3.2, 69.2 ± 2.8, 5.0 ± 3 (n = 3). The proliferation rate of C_2_-ceramide-treated H1299 lung cancer cells significantly decreased in a dose–response manner (P < 0.001). The 50% inhibitory concentration (24 h, IC_50_) of C_2_-ceramide for H1299 cells was 22.9 μM.

### G_1_ arrest of C_2_-Ceramide-treated H1299 lung cancer cells

**Figure 2:**
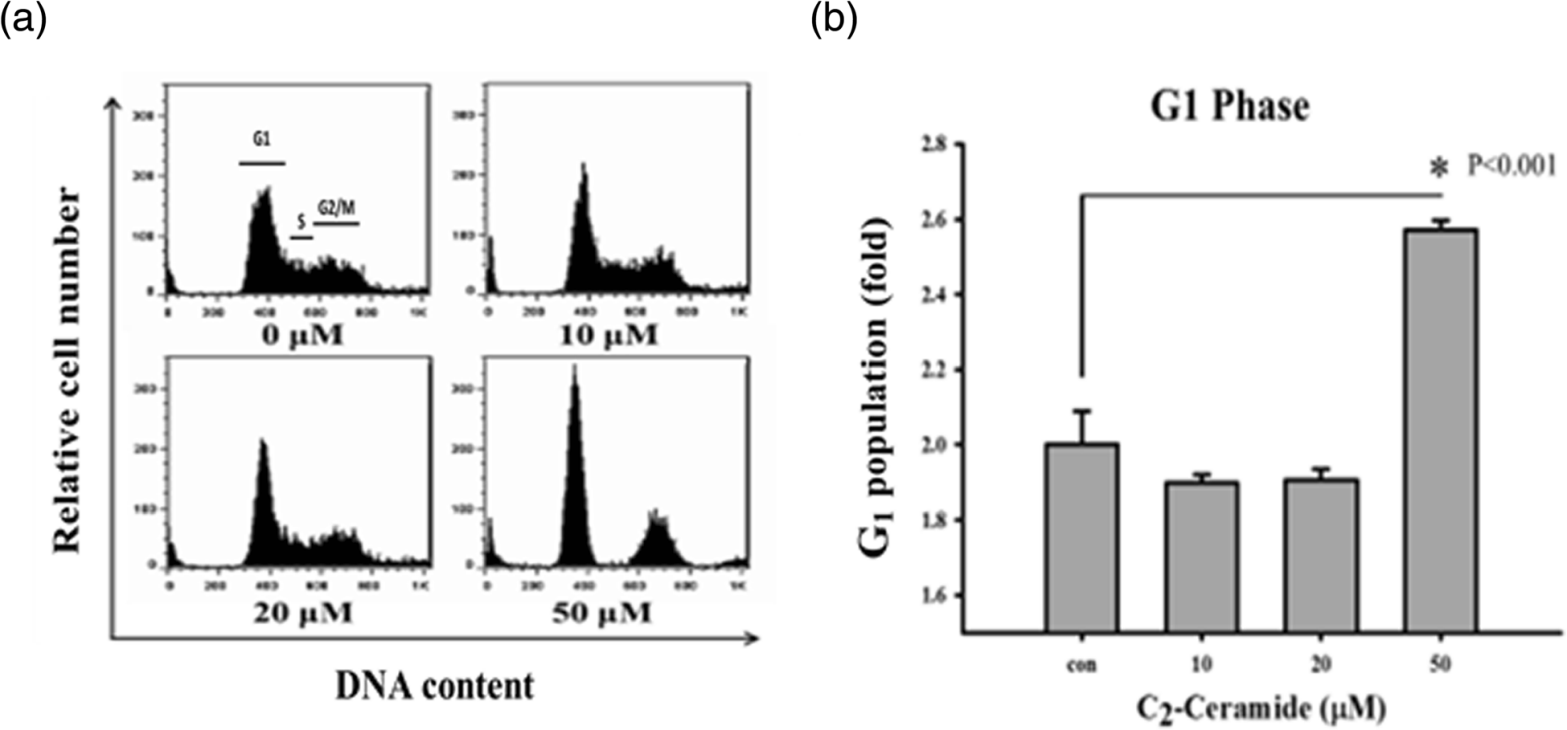
**Treatment with C**_**2**_**-ceramide induced different accumulations of G1 population in lung cancer H1299 cells.** Cells were treated with 0, 10, 20, and 50 μM C_2_-ceramide for 24 h. **(a)** Representative cell cycle distribution in C_2_-ceramide-treated H1299 cells. **(b)** Cell phase percentages obtained for **(a)** in triplicate experiments.

The role of cell cycle interference in the C_2_-ceramide-induced apoptosis of H1299 lung cancer cells was examined by the flow cytometry-based PI assay (Figure 2). The G_1_ percentages were increased at concentration of 50 μM C_2_-ceramide for 24 h (Figure 2a). Apparently, the G_1_ arrest activities in cells treated with C_2_-ceramide showed a significant increase at higher concentration (*P* < 0.001) (Figure 2b).

### Apoptosis induction of C_2_-Ceramide-treated H1299 lung cancer cells

**Figure 3:**
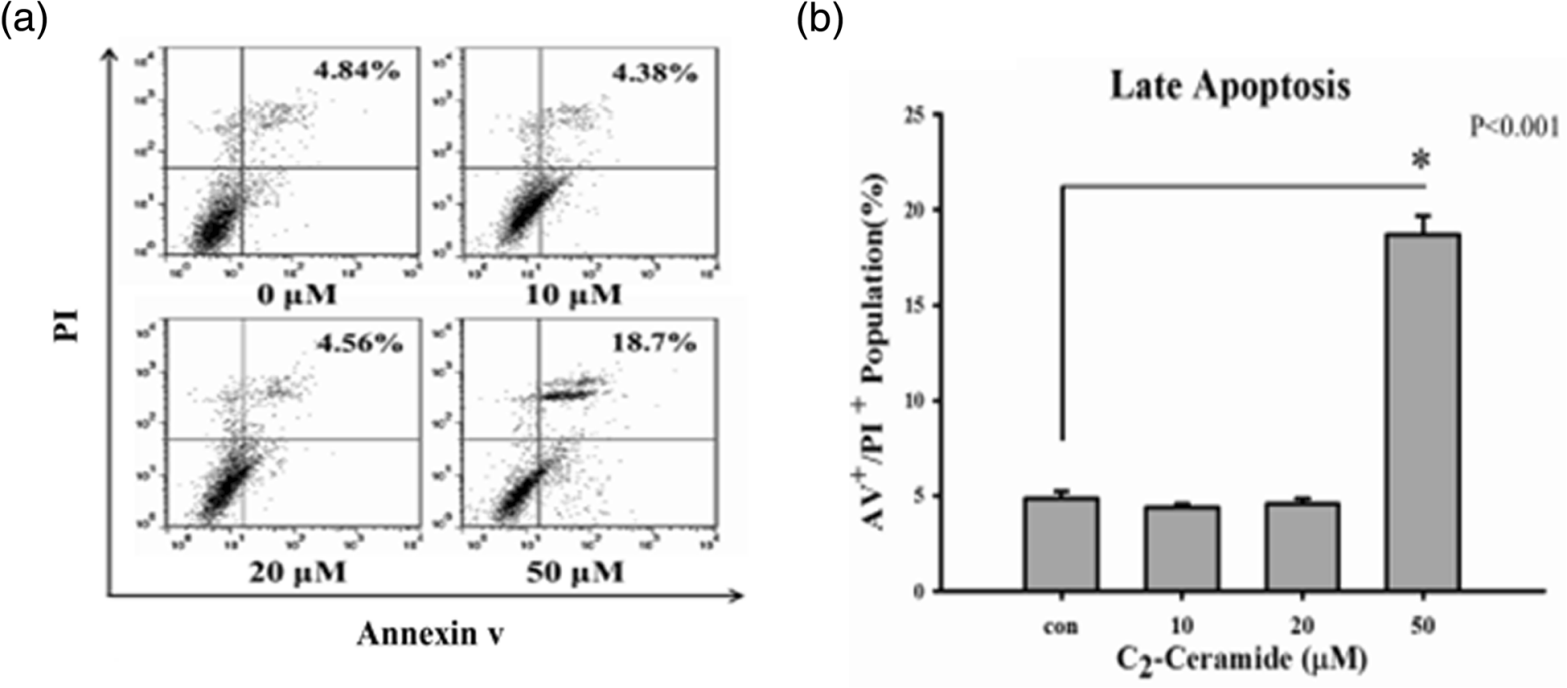
**Treatment with C**_**2**_**-ceramide induced different apoptotic profiles in lung cancer H1299 cells.** Cells were treated with 0, 10, 20, and 50 μM C_2_-ceramide for 24 h. **(a)** Representative apoptotic profiles obtained by Annexin V/PI double staining in C_2_-ceramide-treated H1299 cells. **(b)** Quantification analysis results for late apoptosis population (%). Only annexin V (+)/PI (+) regions were analyzed. Data, mean ± SD (n = 3). Asterisks indicate statistically significant differences compared to control (*P* < 0.001).

In Figure 2a, the profiles of PI/annexin V-positive percentages were shown for the treatments with vehicle control or 0, 10, 20, and 50 μM of C_2_-ceramide for 24 h. After 24 h C_2_-ceramide treatment, the annexin V-positive percentages of H1299 lung cancer cells were significantly increased in a dose–response manner for most concentrations (*P* < 0.05) (Figure 3).

### Chromatin condensation of C_2_-Ceramide-treated H1299 lung cancer cells

**Figure 4:**
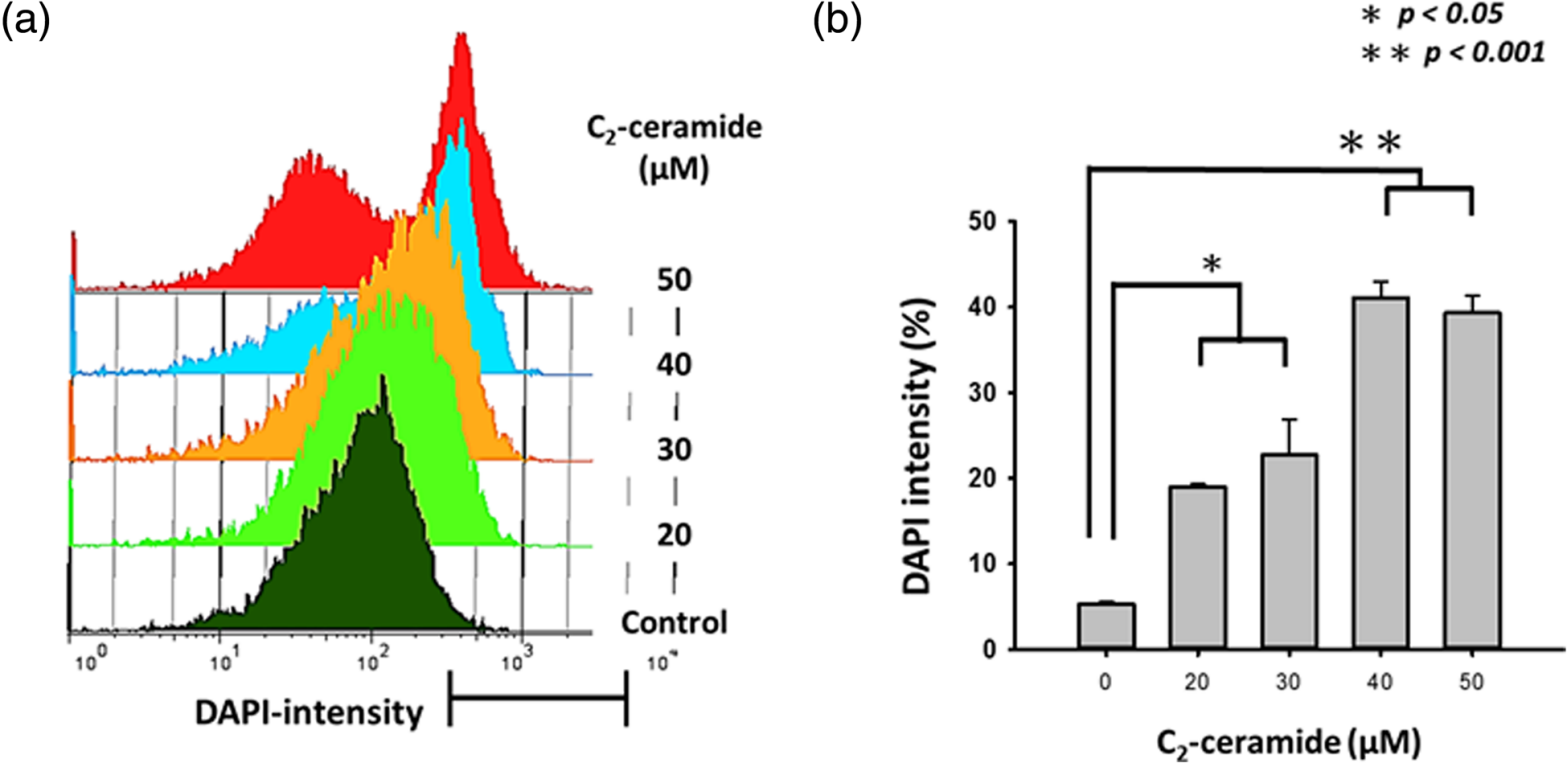
**C**_**2**_**-ceramide increased chromatin condensation levels of H1299 lung cancer cells. (a)** Flow cytometry-based DAPI profiles for C_2_-ceramide-treated cells. Cells treated with different concentrations (0 to 50 μM) of C_2_-ceramide for 24 h. Positive % is indicated in each panel; **(b)** Quantificative analysis of DAPI-positive population. Data are presented as mean ± S.D. (n = 3). Asterisks indicate statistically significant differences compared to control (*P* < 0.001).

Chromatin condensation is one of the most important markers for apoptotic cells [29]. The apoptotic effect of C_2_-ceramide-treated H1299 lung cancer cells was further examined by the flow cytometry-based DAPI assay. The profiles of DAPI-positive percentages of 0, 10, 20, and 50 μM C_2_-ceramide for 24 h were shown (Figure 4a). The DAPI-positive percentages of C_2_-ceramide-treated H1299 lung cancer cells were significantly reduced in a dose–response manner (*P* < 0.001) (Figure 4b).

### Modulation of p-Akt and p-NFκB in C_2_-Ceramide-treated H1299 lung cancer cells

**Figure 5:**
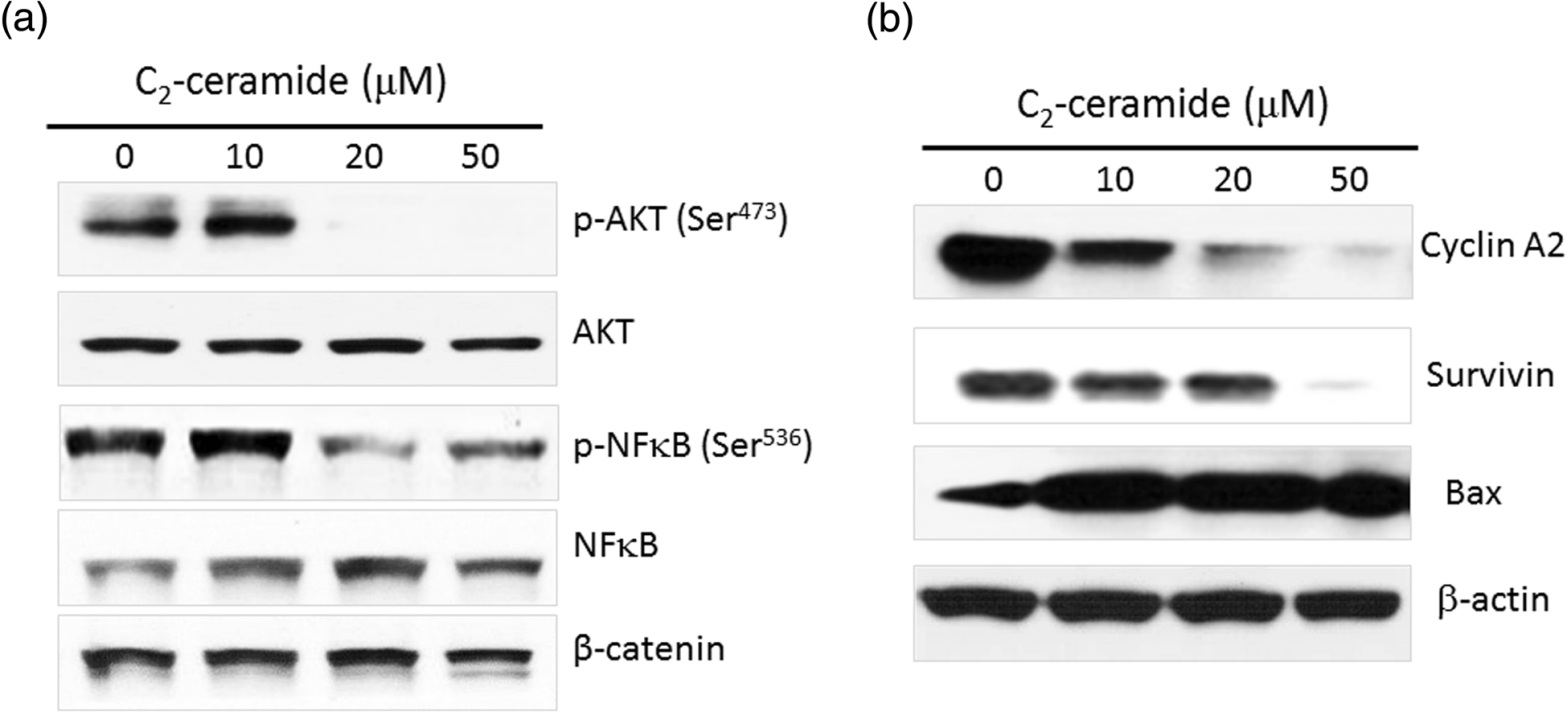
**p-Akt and p-NFκB levels of C**_**2**_**-ceramide-treated H1299 lung cancer cells.** Cells treated with different concentrations (0 to 50 μM) of C_2_-ceramide for 24 h. After treatment, the protein lysates were resolved by SDS-PAGE, transferred onto nitrocellulose membranes and probed with specific antibodies and detected signals using an enhanced chemiluminescence kit. **(a)** The changes of Akt and NFκB phosphorylation. **(b)** The changes of protein level of survivin, cyclin A2 and Bax. β-actin as an internal control.

The role of C_2_-Ceramide-induced modulating the levels of p-Akt and p-NFκB in H1299 lung cancer cells was examined by Western blotting. Both p-Akt and p-NFκB levels in C_2_-Ceramide-treated H1299 cells were significantly reduced at the concentration of 20 and 50 μM (Figure 5a). Likewise, the protein levels of pro-survival survivin and the cell cycle promoter cyclin A2 were down-regulated dramatically. On the contrary, the protein level of pro-apoptotic Bax was increased significantly following C_2_-Ceramide treatment) (Figure 5b).

## Discussion

The modulations of ceramide as the strategy for many kinds of cancer therapies have been reported [18-21]. For example, acid ceramidase was regarded as the target for breast cancer therapy [19] because it can hydrolyze ceramide, and thus reduce its intracellular levels. Previous study showed that C_2_-ceramide is not very harmful to normal cells. For example, the IC_50_ (24 h) of human dermal neonatal fibroblast (HDNF) cells for C_2-_ceramide was 66.5 μM [30], suggesting the moderately selective anti-proliferative effect of C_2-_ceramide toward cancer cells. In the current study, we found that the C_2_-ceramide induced apoptosis of H1299 lung cancer cells. It provides the idea that pharmacological modulation of sphingolipid metabolism can enrich the tumor cell ceramide for cancer chemotherapy.

Sometimes the degree of the sub-G_1_ accumulation may not appear in concert with the apoptosis in terms of Annexin V/PI staining. For example, no sub-G_1_ accumulation was found in C_2_-ceramide-treated H1299 lung cancer cells at 24 h treatment, but it still showed the apoptosis-inducible effects in terms of Annexin V/PI and DAPI-based chromatin condensation assays using flow cytometry. Similarly, (-)-Anonaine inhibits growth of H1299 cells without sub-G_1_ accumulation before 48 h incubation, however, the (-)-Anonaine ends up increasing apoptosis at 72 h treatment [31]. Therefore, the absence of sub-G_1_ accumulation in C_2_-ceramide-treated H1299 at 24 h treatment may be due to the detection timing. Furthermore, chromatin condensation was thought to be one of hallmarks in apoptotic cells [32,33]. However, some study indicated that certain stresses such as heat shock may induce a non-apoptotic chromosome condensation [34]. For example, Plehn-Dujowich’s work found the non-apoptotic chromatin condensation [34]. Accordingly, in our study, the non-apoptosis inducing dose of 20 μM C_2_-ceramide caused stress-induced chromatin condensation, which may explain the reason that C_2_-ceramide induces anti-proliferation without apoptosis (Figure 4). Eventually, a higher dose of 50 μM C_2_-ceramide causes the apoptotic chromatin condensation, resulting in cell death of H1299 cells.

**Figure 6:**
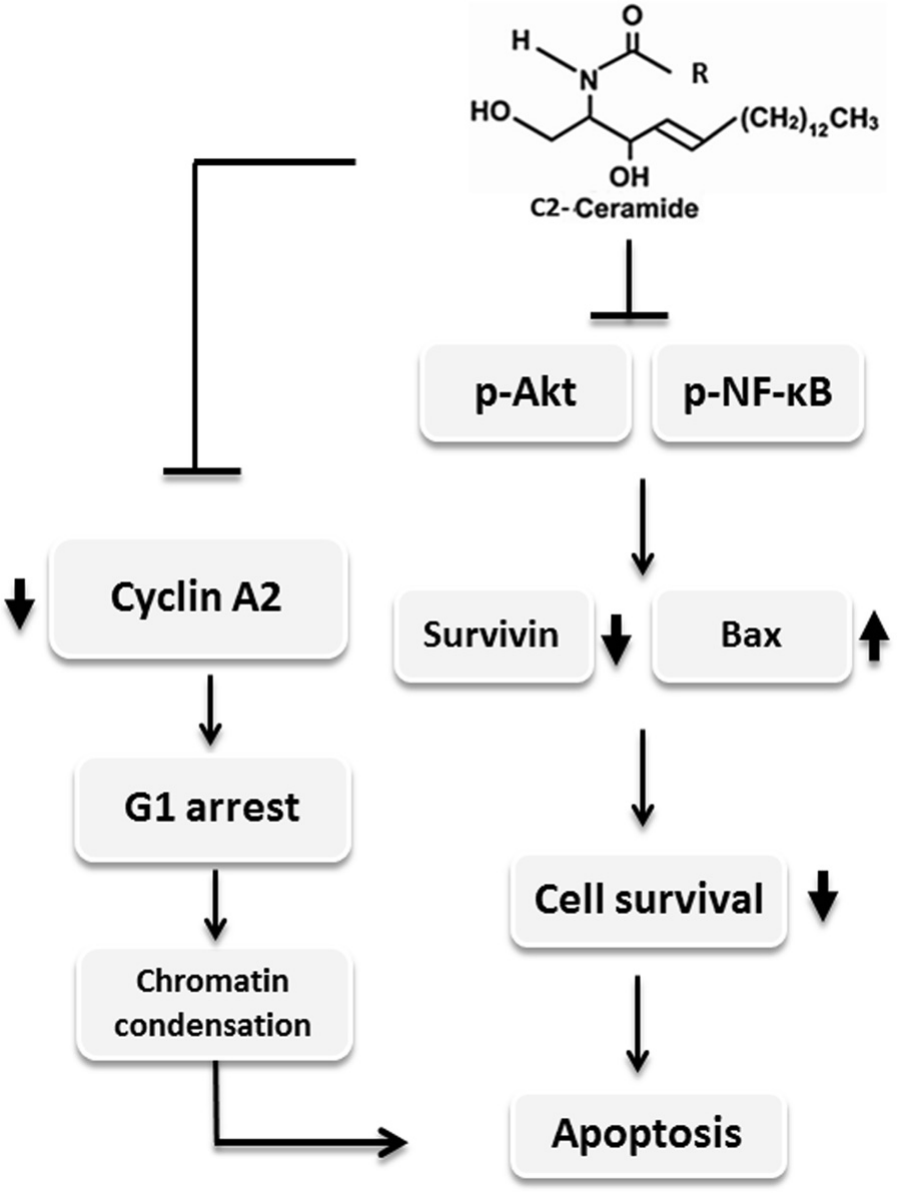
**Schematic diagram of hypothesized mechanism of C**_**2**_**-ceramide-induced apoptosis of lung cancer cells.** C_2_-ceramide inhibits the activity of both Akt and NF-κB, causing the down-regulation of pro-survival survivin and cell cycle promoter cyclin A2. On the contrary, C_2_-ceramide increases the protein level of pro-apoptotic Bax. As a result, C_2_-creamide treatment causes cell cycle G_1_ arrest and chromatin condensation, subsequently, triggering the apoptosis of lung cancer cells.

Previously, C_2_-ceramide-induced H1299 cells was investigated [22,23]. Demarchi’s work indicated that C_2_-ceramide triggers the NFκB-dependent survival pathway. However, our study showed that C_2_-ceramide dramatically decreases the level of phosphorylated NFκB (Figure 5a). This may due to the different duration of NFκB treatment (8 h *vs*. 24 h for Demarchi and Lin respectively). Importantly, our study demonstrated that C_2_-ceramide potently inhibits Akt phosphorylation of H1299 cells at the of 20 and 50 μM, suggesting that it will be an advantage of treating lung cancer with constitutively phosphorylated Akt. C_2_-ceramide also causes the down-regulation of survivin and cyclin A2 (Figure 5b), and the up-regulation of pro-apoptotic factor Bax in H1299 cells. This may sensitize lung cancer cells towards proliferation inhibition and apoptosis (Figure 6). The results of this study demonstrated that that C_2_-ceramide treatment exerts anti-growth potential against human non-small cell lung cancer cells H1299 in a dose-responsive manner. C_2_-ceramide also reduces the pro-survival proteins Akt and NFκB, causing the down-regulation of survivin and cyclin A2, which are reported to frequently overexpress in non-small cell lung cancer [35]. This may sensitize lung cancer cells towards proliferation inhibition and apoptosis (Figure 6). Accordingly, the above results suggested that C_2_-ceramide may be a promising reagent for lung cancer treatment or adjuvant therapy in future.

## Competing interest

The authors declare no conflict of interest.

## Author’s contribution

Conceived and designed the experiments: HLC, HMW and ILL; Performed experiments: HLC, JCL and FWC; Data analysis: YH and HWH; Contributed reagents/materials: WCC, CYW and WTC; Manuscript writing: CCC and ILL. All authors have read and approved the manuscript.

## References

[B1] Travis WD Brambilla E Noguchi M Nicholson AG Geisinger K Yatabe Y Powell CA Beer D Riely G Garg K International association for the study of lung cancer/American thoracic society/European respiratory society: international multidisciplinary classification of lung adenocarcinoma: executive summary Proceedings of the American Thoracic Society 2011 8 5 381 385 10.1513/pats.201107-042ST 21926387

[B2] Hsu HS Chen CY Lee CF Wang YC* The tobacco-specific carcinogen NNK induces DNA methyltransferase 1 accumulation and tumor suppressor gene hypermethylation in mice and lung cancer patients J Clin Invest 2010 120 521 532 10.1172/JCI40706 20093774 PMC2810088

[B3] Sun S Schiller JH Gazdar AF Lung cancer in never smokers–a different disease Nature reviews Cancer 2007 7 10 778 790 10.1038/nrc2190 17882278

[B4] Herbst RS Heymach JV Lippman SM Lung cancer N Engl J Med 2008 359 13 1367 1380 10.1056/NEJMra0802714 18815398 PMC10662965

[B5] Shrivastav A Murphy L Interactions of PI3K/Akt/mTOR and estrogen receptor signaling in breast cancer Breast Cancer Management 2012 1 3 235 249 10.2217/bmt.12.37

[B6] Hafner C Landthaler M Vogt T Activation of the PI3K/AKT signalling pathway in non-melanoma skin cancer is not mediated by oncogenic PIK3CA and AKT1 hotspot mutations Experimental dermatology 2010 19 8 e222 227 10.1111/j.1600-0625.2009.01056.x 20557351

[B7] Saitoh Y Martinez Bruyn VJ Uota S Hasegawa A Yamamoto N Imoto I Inazawa J Yamaoka S Overexpression of NF-kappaB inducing kinase underlies constitutive NF-kappaB activation in lung cancer cells Lung cancer 2010 70 3 263 270 10.1016/j.lungcan.2010.03.001 20338663

[B8] Tang X Liu D Shishodia S Ozburn N Behrens C Lee JJ Hong WK Aggarwal BB Wistuba II Nuclear factor-kappaB (NF-kappaB) is frequently expressed in lung cancer and preneoplastic lesions Cancer 2006 107 11 2637 2646 10.1002/cncr.22315 17078054

[B9] Gowda R Madhunapantula SV Desai D Amin S Robertson GP Simultaneous targeting of COX-2 and AKT using selenocoxib-1-GSH to inhibit melanoma Molecular cancer therapeutics 2013 12 1 3 15 10.1158/1535-7163.MCT-12-0492 23112250 PMC3546139

[B10] Yeramian A Sorolla A Velasco A Santacana M Dolcet X Valls J Abal L Moreno S Egido R Casanova JM Inhibition of activated receptor tyrosine kinases by Sunitinib induces growth arrest and sensitizes melanoma cells to Bortezomib by blocking Akt pathway International journal of cancer Journal international du cancer 2012 130 4 967 978 10.1002/ijc.26096 21445974

[B11] Chen W Li Z Bai L Lin Y NF-kappaB in lung cancer, a carcinogenesis mediator and a prevention and therapy target Frontiers in bioscience 2011 16 1172 1185 10.2741/3782 PMC3032584 21196225

[B12] Jin X Qiu L Zhang D Zhang M Wang Z Guo Z Deng C Guo C Chemosensitization in non-small cell lung cancer cells by IKK inhibitor occurs via NF-kappaB and mitochondrial cytochrome c cascade Journal of cellular and molecular medicine 2009 13 11–12 4596 4607 19067767 10.1111/j.1582-4934.2008.00601.x PMC4515074

[B13] Schuchman EH Acid sphingomyelinase, cell membranes and human disease: lessons from Niemann-Pick disease FEBS letters 2010 584 9 1895 1900 10.1016/j.febslet.2009.11.083 19944693

[B14] Venable ME Yin X Ceramide induces endothelial cell senescence Cell biochemistry and function 2009 27 8 547 551 10.1002/cbf.1605 19842094

[B15] Huang WC Chen CL Lin YS Lin CF Apoptotic sphingolipid ceramide in cancer therapy J Lipids 2011 2011 565316 21490804 10.1155/2011/565316 PMC3066853

[B16] Kim HJ Oh JE Kim SW Chun YJ Kim MY Ceramide induces p38 MAPK-dependent apoptosis and Bax translocation via inhibition of Akt in HL-60 cells Cancer letters 2008 260 1–2 88 95 18054155 10.1016/j.canlet.2007.10.030

[B17] Zhou H Summers SA Birnbaum MJ Pittman RN Inhibition of Akt kinase by cell-permeable ceramide and its implications for ceramide-induced apoptosis J Biol Chem 1998 273 26 16568 16575 10.1074/jbc.273.26.16568 9632728

[B18] Morad SA Messner MC Levin JC Abdelmageed N Park H Merrill AH Jr Cabot MC Potential role of acid ceramidase in conversion of cytostatic to cytotoxic end-point in pancreatic cancer cells Cancer chemotherapy and pharmacology 2013 71 3 635 645 10.1007/s00280-012-2050-4 23263160

[B19] Flowers M Fabrias G Delgado A Casas J Abad JL Cabot MC C6-ceramide and targeted inhibition of acid ceramidase induce synergistic decreases in breast cancer cell growth Breast cancer research and treatment 2012 133 2 447 458 10.1007/s10549-011-1768-8 21935601

[B20] Huang H Zhang Y Liu X Li Z Xu W He S Huang Y Zhang H Acid sphingomyelinase contributes to evodiamine-induced apoptosis in human gastric cancer SGC-7901 cells DNA and cell biology 2011 30 6 407 412 10.1089/dna.2010.1122 21294641

[B21] Fabrias G Bedia C Casas J Abad JL Delgado A Ceramidases in hematological malignancies: senseless or neglected target? Anti-cancer agents in medicinal chemistry 2011 11 9 830 843 10.2174/187152011797655104 21707488

[B22] Demarchi F Bertoli C Greer P Schneider C Ceramide triggers an NF-κB-dependent survival pathway through calpain Cell Death & Differentiation 2005 12 5 512 522 10.1038/sj.cdd.4401592 15933726

[B23] Xu L Deng X Suppression of cancer cell migration and invasion by protein phosphatase 2A through dephosphorylation of μ-and m-calpains Journal of Biological Chemistry 2006 281 46 35567 35575 10.1074/jbc.M607702200 16982626

[B24] Xu J Qian J Xie X Lin L Zou Y Fu M Huang Z Zhang G Su Y Ge J High density lipoprotein protects mesenchymal stem cells from oxidative stress-induced apoptosis via activation of the PI3K/Akt pathway and suppression of reactive oxygen species International journal of molecular sciences 2012 13 3 17104 17120 23443132 10.3390/ijms131217104 PMC3546741

[B25] Yen CY Chiu CC Chang FR Chen JY Hwang CC Hseu YC Yang HL Lee AY Tsai MT Guo ZL 4beta-Hydroxywithanolide E from *Physalis peruviana* (golden berry) inhibits growth of human lung cancer cells through DNA damage, apoptosis and G2/M arrest BMC Cancer 2010 10 46 10.1186/1471-2407-10-46 20167063 PMC2830937

[B26] Chiu CC Chang HW Chuang DW Chang FR Chang YC Cheng YS Tsai MT Chen WY Lee SS Wang CK Fern plant-derived protoapigenone leads to DNA damage, apoptosis, and G(2)/m arrest in lung cancer cell line H1299 DNA and cell biology 2009 28 10 501 506 10.1089/dna.2009.0852 19630532

[B27] Chiu CC Haung JW Chang FR Huang KJ Huang HM Huang HW Chou CK Wu YC Chang HW Golden berry-derived 4beta-hydroxywithanolide E for selectively killing oral cancer cells by generating ROS, DNA damage, and apoptotic pathways PLoS One 2013 8 5 e64739 10.1371/journal.pone.0064739 23705007 PMC3660349

[B28] Yen CY Chiu CC Haung RW Yeh CC Huang KJ Chang KF Hseu YC Chang FR Chang HW Wu YC Antiproliferative effects of goniothalamin on Ca9-22 oral cancer cells through apoptosis; DNA damage and ROS induction Mutation research 2012 747 2 253 258 10.1016/j.mrgentox.2012.06.003 22721813

[B29] Oberhammer FA Hochegger K Froschl G Tiefenbacher R Pavelka M Chromatin condensation during apoptosis is accompanied by degradation of lamin A + B, without enhanced activation of cdc2 kinase The Journal of cell biology 1994 126 4 827 837 10.1083/jcb.126.4.827 8051209 PMC2120132

[B30] Kang J-H Garg H Sigano DM Francella N Blumenthal R Marquez VE Ceramides: Branched alkyl chains in the sphingolipid siblings of diacylglycerol improve biological potency Bioorganic & Medicinal Chemistry 2009 17 4 1498 1505 10.1016/j.bmc.2009.01.005 19171486 PMC6980351

[B31] Chen BH Chang HW Huang HM Chong IW Chen JS Chen CY Wang HM (-)-Anonaine induces DNA damage and inhibits growth and migration of human lung carcinoma H1299 cells J Agric Food Chem 2011 59 6 2284 2290 10.1021/jf103488j 21361287

[B32] La Vignera S Condorelli R Vicari E D’Agata R Calogero AE Effects of varicocelectomy on sperm DNA fragmentation, mitochondrial function, chromatin condensation, and apoptosis J Androl 2012 33 3 389 396 10.2164/jandrol.111.013433 21636732

[B33] Fang M Zhang HQ Xue SB Apoptosis of HL-60 cells induced by Harringtonine: membrane blebs, nucleus blebs and chromatin condensation Shi Yan Sheng Wu Xue Bao 1996 29 3 221 233 9639809

[B34] Plehn-Dujowich D Bell P Ishov AM Baumann C Maul GG Non-apoptotic chromosome condensation induced by stress: delineation of interchromosomal spaces Chromosoma 2000 109 4 266 279 10.1007/s004120000073 10968255

[B35] Ko E Kim Y Cho EY Han J Shim YM Park J Kim D-H Synergistic effect of Bcl-2 and Cyclin A2 on adverse recurrence-free survival in stage i non-small cell lung cancer Annals of surgical oncology 2013 20 1005 1012 10.1245/s10434-012-2727-2 23115005

